# Proximal vs. Recipient Site for Vascular Lymph Node Transfers for Breast Cancer-Related Lymphedema: A Meta-Analysis and Systematic Review

**DOI:** 10.3390/jcm14207281

**Published:** 2025-10-15

**Authors:** Jenna-Lynn B. Senger, Ramin Rajaii, Christopher Slater, Min-Jeong Cho

**Affiliations:** 1 Division of Plastic Surgery, Vancouver Coastal Health, Vancouver General Hospital, 1788-1111 West Georgia Street, Vancouver, BC V6E 4M3, Canada; jenna.senger@ubc.ca; 2 Department of Plastic and Reconstructive Surgery, The Ohio State University Wexner Medical Center, 915 Olentangy River Road, 2nd Floor Suite 2140, Columbus, OH 43212, USA; ramin.rajaii@osumc.edu (R.R.); christopher.slater@osumc.edu (C.S.)

**Keywords:** lymphedema, breast cancer, vascularized lymph node transfer, breast cancer-related lymphedema, BCRL, VLNT

## Abstract

**Background/Objectives**: Despite the popularity of vascularized lymph node transfers (VLNTs) for treatment of breast cancer-related lymphedema (BCRL), the comparative effectiveness of VLNT placement locations is unknown. In this meta-analysis, we examined the impact of VLNT recipient site (proximal vs. distal vs. dual) and adjunct surgical techniques, including scar release, supercharging, and intervention timing on patient outcomes. **Methods**: PRISMA-guided search of PubMed, MEDLINE, and Embase (January 2015–March 2025). Patient outcomes including limb circumference/volume reduction, cellulitis frequency, compression garment discontinuation, and patient satisfaction were analyzed. Subgroup analyses assessed node placement, scar release, supercharging, and timing. **Results**: A total of 1440 patients were analyzed (proximal 63.8%; distal 29.2%; dual 7.0%). No significant differences in mean volume/circumference reductions, patient satisfaction rates, and compression garment discontinuation were observed amongst placement strategies (*p* > 0.30). Adjunct scar release (65%) was associated with significantly greater patient satisfaction (*p* = 0.04) and showed trends toward improved volume reduction and compression discontinuation. Earlier intervention (<5 years from diagnosis) showed improved volume reduction, patient satisfaction, and compression discontinuation. Longitudinal analysis revealed dual placement maintained superiority throughout a 12-month follow-up. **Conclusions**: VLNT is an effective treatment for BCRL regardless of placement location, with all strategies demonstrating substantial clinical improvements. While dual placement showed numerically superior outcomes across all measures, differences did not reach statistical significance due to limited number of studies. Adjunct scar release significantly improves patient satisfaction, and earlier intervention may optimize outcomes. These findings suggest that recipient site selection may be guided by technical factors, vessel availability, and patient preference rather than efficacy differences.

## 1. Introduction

Breast cancer is the most frequently diagnosed cancer among women worldwide, with an estimated 2.3 million new cases and 670,000 deaths in 2022 [1]. Advances in treatment have improved 5-year survival to around 90%, yielding a growing population of survivors at risk for long-term complications [WHO forecast]. One such complication is breast cancer-related lymphedema (BCRL), a chronic, debilitating condition of arm swelling resulting from lymphatic injury due to surgery and/or radiation. Studies report incidence rates of BCRL ranging from single digits to as high as 30–40% [2]. This condition negatively impacts quality of life with symptoms of limb heaviness, recurrent infections (cellulitis), pain, and functional impairment [3]. Conservative therapies (e.g., compression garments, manual lymphatic drainage, and complex decongestive therapy) are first-line and can ameliorate early-stage lymphedema, but many patients with moderate-to-severe BCRL have persistent swelling and morbidity despite maximal non-surgical management.

In recent years, microsurgical approaches have emerged to restore lymphatic drainage in BCRL. Two primary physiologic surgical interventions are lymphovenous bypass (LVB)—connecting lymphatic vessels to veins—and vascularized lymph node transfer (VLNT), in which a free tissue flap containing functional lymph nodes is transplanted to the affected limb [4]. VLNT aims to re-establish lymphatic flow or stimulate lymphangiogenesis by introducing healthy lymph nodes with their blood supply into the lymphedematous limb. In a VLNT procedure, lymph node flaps (e.g., from inguinal, submental, or supraclavicular basins) are harvested on their vascular pedicle and transferred to the arm, where microsurgical anastomoses to recipient blood vessels maintain flap perfusion [5,6]. VLNT is often reserved for patients with BCRL who have failed LVB or conservative therapy; it can also be performed concurrently with LVB or autologous breast reconstruction in select cases to maximize therapeutic benefit [1]. Given the complexity and resource-intensive nature of VLNT, optimizing surgical technique and patient selection is critical to improving outcomes.

One of the most debated aspects of VLNT is recipient site selection. There remain no consensus guidelines identifying whether the lymph nodes should be inset proximally in the axilla or distally in the forearm/wrist [5,6]. The physiologic rationale for proximal (“orthotopic transfer”) and distal (“heterotopic transfer”) node placement is rooted in differing mechanisms of lymphatic modulation based on the lymphatic drainage dynamics and the anatomic characteristics of the lymphedematous limb [7]. Proximal node placement is thought to directly address the primary site of lymphatic obstruction, while distal placement places the transplanted nodes directly in the region of pooling [5]. Although proponents of both techniques have cited theoretical and clinical advantages, few studies have directly compared proximal vs. distal recipient sites, and those that do exist are generally limited by small sample sizes [8].

More recently, “dual placement” VLNT, in which lymph node flaps are placed both proximally and distally, has also been described as a strategy to leverage the benefits of both approaches [9,10]. Understanding the physiological mechanisms, lymphedema outcomes, and associated clinical benefits/detriments of each approach is crucial for optimizing surgical techniques to improve patient outcomes.

Despite growing interest in recipient site optimization, there remains a lack of consensus on best practices. A prior meta-analysis published in 2022 summarized outcomes of proximal and distal VLNTs based on the literature through 2020, with no obvious superiority between sites identified [11]. Nearly four years have passed since this review, and there remains no universally agreed upon recipient site for VLNTs in BCRL. As VLNTs become increasingly integrated into lymphedema care, the number of publications reporting clinical outcomes has expanded. In this review, we re-evaluate the updated literature to better ascertain the optimal location for VLNT placement: proximal or distal.

## 2. Materials and Methods

### 2.1. Protocol and Registration

This systematic review and meta-analysis was conducted in accordance with the PRISMA 2020 guidelines for Preferred Reporting Items for Systematic Reviews and Meta-Analyses. The review protocol was not registered on PROSPERO or any other registry, and no prior protocol document was prepared (PRISMA item 24a/24b).

### 2.2. Eligibility Criteria

We defined inclusion and exclusion criteria a priori following the PICOS framework (Population, Intervention, Comparison, Outcomes, Study design). Eligible studies were clinical studies (randomized or non-randomized) that reported outcomes of VLNTs performed specifically for upper-extremity BCRL in human patients. This included randomized controlled trials, cohort and case-control studies, observational studies, and larger case series. To focus on comparative effectiveness, studies had to report quantitative clinical outcomes after VLNT (such as limb circumference or volume change, infection rates, etc.). We excluded publications not meeting these criteria or not isolating BCRL patients.

Exclusion criteria were as follows: studies on animal models; non-original papers (reviews, commentaries, letters); single-patient case reports; studies focusing on primary (non-cancer) or lower-extremity lymphedema; series where VLNT was combined with other surgical interventions (e.g., liposuction or LVB) without separate analysis of VLNT effects; and studies in which outcomes of VLNT for BCRL could not be distinguished from other interventions or populations. If a study included mixed etiologies or adjunct procedures, we included it only if data specific to BCRL VLNT could be extracted. There were no restrictions on language (except that non-English papers were removed due to practical screening limitations) or minimum sample size. Studies from 1 January 2015 up to 1 March 2025 were considered to ensure contemporary surgical techniques were represented.

### 2.3. Information Sources and Search Strategy

A comprehensive literature search was performed in April 2025 using the following databases: PubMed (MEDLINE), Ovid MEDLINE, and Embase. The search strategy combined terms for lymph node transfer and lymphedema and was developed with PRISMA-S guidance. Specifically, we used keywords and MeSH terms including: “(lymph node OR lymph-node) AND (transfer *OR transplant* OR flap) AND (lymphedema OR lymphoedema)”. The search was filtered to the publication date range 2015–2025 and human studies; no geographic or language limits were applied initially (non-English papers were later excluded during screening). We also manually reviewed the reference lists of relevant articles and prior reviews to identify any additional studies. The final search was conducted on May 2025, and the full detailed strategies for each database are provided in Supplementary Table S1 (PRISMA item 7). All search results were imported into a reference manager, and duplicate records were identified and removed before screening.

### 2.4. Study Selection (Screening and Exclusion Process)

Two reviewers (C.S. and R.R.) independently screened the titles and abstracts of all unique records for potential relevance (PRISMA item 8). Studies clearly not meeting inclusion criteria (e.g., studies on unrelated topics, non-BCRL lymphedema, or non-VLNT interventions) were excluded at this stage. After title/abstract screening of 844 unique records, 690 studies were excluded as irrelevant or ineligible. The most common reasons for exclusion were off-topic or wrong patient population (e.g., lymphedema not related to breast cancer, or animal/basic science studies), inappropriate publication type (case reports, reviews, editorials without original data), or inadequate outcomes data (e.g., did not report limb outcomes specific to VLNT).

Next, the full texts of the remaining 154 potentially eligible studies were retrieved for detailed review by two reviewers working independently (C.S. and R.R.). During full-text eligibility assessment, an additional 117 studies were excluded. Key reasons for full-text exclusion included mixed interventions or populations without separable data (for example, VLNT combined with liposuction or LVB in a way that outcomes for VLNT alone could not be isolated), outcomes not clearly reported or quantified (e.g., papers describing surgical technique with only qualitative outcomes), duplicate or overlapping patient cohorts (more than one study reporting on the same patient series—in such cases the most comprehensive or recent report was included), and failure to focus on BCRL (some studies included multiple lymphedema etiologies but did not segregate breast cancer subset results).

**Figure 1:**
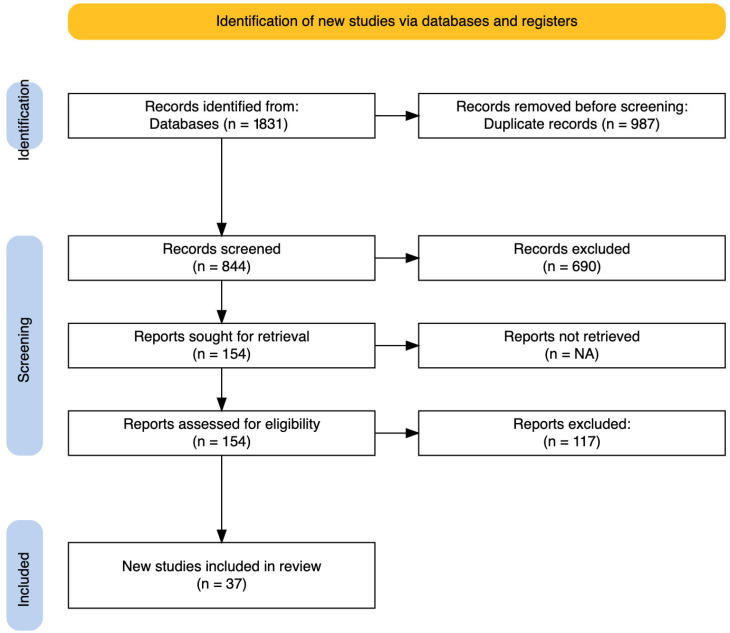
PRISMA 2020 flow diagram for systematic review of the literature.

Any disagreements in study inclusion between the two screeners were resolved by discussion or by consulting a third author (J.L.S.) as needed. Figure 1 shows the PRISMA 2020 flow diagram illustrating the study selection process, including the number of records identified, screened, excluded, and ultimately included in the review.

In total, 37 studies met all criteria and were included in the final qualitative and quantitative synthesis (PRISMA item 16a).

### 2.5. Data Collection and Extraction

A standardized data extraction form was used to collect pertinent data from each included study (PRISMA item 9). Two reviewers (R.R. and C.S.) independently extracted the data, which were then cross-verified. The following data items were collected from each report: study characteristics (first author, publication year, country, study design, sample size); patient population details (mean age, body mass index, cancer treatment factors such as radiation history, lymphedema stage if reported, and mean duration of lymphedema or time from cancer diagnosis to VLNT); and surgical details of the VLNT procedure (donor lymph node site used, recipient site location—proximal axilla, distal wrist/forearm, or dual—the recipient vessels for anastomosis, and the microsurgical techniques used such as end-to-end vs. end-to-side anastomoses or flow-through flap design).

We also recorded whether certain adjunct measures were employed, e.g., performance of axillary scar release (surgical release of scar tissue or capsular contracture in the axilla, if reported), use of concurrent non-VLNT flaps (such as combined breast reconstruction flaps), use of postoperative manual lymphatic drainage (MLD) therapy, and whether the VLNT was “supercharged” (defined as additional venous anastomoses or other modifications to augment flap outflow). If not explicitly stated, we inferred adjuncts from methods (for instance, if authors described releasing scar tissue or adding a vein anastomosis). Any clarifications needed were resolved by consensus among the authors.

### 2.6. Outcomes Assessed

The primary outcome measures extracted for each study were the improvements in limb lymphedema and related clinical endpoints (PRISMA item 10a). Specifically, we recorded arm circumference reduction (CR), often reported as reduction in excess circumference or percentage reduction in circumference difference between limbs; excess limb volume reduction (EVR), a reduction in excess volume of the affected arm compared to the healthy arm, often given as a percentage; frequency of cellulitis/infection episodes per year before and after VLNT; discontinuation of compression garments (proportion of patients able to stop using compression sleeves after VLNT); and patient-reported outcomes or satisfaction, such as the percentage of patients reporting subjective improvement in swelling or overall satisfaction with treatment. We also extracted follow-up duration for outcomes (mean or median follow-up time after surgery, in months) for each study, to assess how long outcomes were measured.

We collected complications data, classifying complications as minor or major. We adopted the Clavien–Dindo classification where possible: minor complications were those not requiring invasive intervention (e.g., seroma, minor wound issues), whereas major complications were those of Grade ≥ III (requiring surgical re-intervention or causing significant morbidity). In particular, we noted any instances of flap failure (complete flap loss due to thrombosis or necrosis), which would be a major complication. If a study did not explicitly categorize complications, we recorded any described events (e.g., infections, reoperations, donor site issues) and later grouped them as minor/major during analysis.

### 2.7. Risk of Bias and Quality Assessment

We did not perform a formal risk-of-bias assessment for each study (PRISMA item 11). Given the diverse study designs (including only 2 randomized trials among mostly observational studies) and the lack of a uniformly applicable quality appraisal tool for this mix, we chose to focus on quantitative synthesis of available data. We acknowledge this as a limitation. In lieu of a numeric risk-of-bias score, we qualitatively noted potential sources of bias: most included studies were retrospective case series with inherent selection bias (surgeons chose which patients and techniques to use) and reporting bias and often lacked control groups. The two randomized trials had small sample sizes and risk of performance bias (lack of blinding). No automation tools were used for bias assessment.

### 2.8. Data Synthesis and Statistical Analysis

We performed a meta-analysis of pooled outcomes using standard methods (PRISMA items 13a–13d). For each outcome measure (e.g., percent volume reduction), summary statistics from each study were compiled. Where possible, we calculated pooled mean differences or pooled proportions with 95% confidence intervals. Given anticipated inter-study heterogeneity, a random-effects model (DerSimonian–Laird method) was used for meta-analytic pooling of continuous and proportion outcomes. All meta-analyses were conducted using R software (v4.2.2) with the *meta* package. For continuous outcomes reported in different units or baseline references, we used mean percentage reduction as the common effect measure to allow comparison across studies (e.g., studies reporting absolute volume change were converted to % reduction from baseline when possible). Heterogeneity was assessed with the Cochran’s Q test and quantified by the I^2^ statistic, with I^2^ > 50% indicating substantial heterogeneity. We also inspected forest plots for overlap of confidence intervals. Subgroup analyses were defined a priori to explore potential sources of heterogeneity (PRISMA item 13e).

We stratified outcomes by VLNT recipient site (proximal vs. distal vs. dual placement) to compare their effectiveness. We also performed subgroup analyses based on surgical adjuncts: comparing outcomes in patients who had axillary scar release vs. those who did not, supercharged VLNTs vs. conventional VLNTs, and patients treated earlier (<5 years from cancer diagnosis) vs. those with longer-standing lymphedema. These subgroup analyses were conducted when at least 3 studies or ≥50 patients were available in each subgroup; results were considered hypothesis-generating given the observational data. We did not conduct formal sensitivity analyses (PRISMA 13f) other than using an alternate pooling method (fixed effect) to check consistency.

### 2.9. Use of Generative AI and Software

Analyses and figures were performed in R (v4.2.2). Claude Opus 4.1 (Anthropic) was used solely to assist with debugging R code and optimizing computational efficiency. All statistical methods, study parameters, and figure specifications were determined by the human authors; all outputs were independently verified prior to inclusion. No generative AI was used for study conception or design, literature screening/selection, data extraction, interpretation of results, or manuscript writing/editing. This disclosure follows JCM/MDPI author guidance for reporting GenAI use in the Methods.

### 2.10. Publication Bias

We assessed potential publication bias by examining funnel plots for the primary outcomes (volume and circumference reduction) (PRISMA item 14). The funnel plots were visually inspected for asymmetry, and we used Egger’s regression test to statistically test for asymmetry since ≥10 studies were included in the meta-analysis. No formal certainty of evidence (GRADE) evaluation was performed (PRISMA item 15), given that most data were level IV evidence.

### 2.11. PRISMA Checklist Compliance

We ensured that all relevant PRISMA 2020 checklist items were addressed in our methods and reporting. Table S1 in the Supplementary Materials provides a summary mapping of each PRISMA item in the Materials and Methods to where it is reported in this manuscript for transparency.

## 3. Results

### 3.1. Search Methodology

A total of 1831 articles were identified in the initial search, with 37 studies meeting inclusion criteria for final analysis [5,6,12,13,14,15,16,17,18,19,20,21,22,23,24,25,26,27,28,29,30,31,32,33,34,35,36,37,38,39,40,41,42,43,44]. Of the included studies, 14 were prospective (38%) and 23 retrospective (62%). Study types included randomized controlled trials (*n* = 2), cohort studies (*n* = 15), observational studies (*n* = 12), and case series (*n* = 8).

### 3.2. Patient Demographics and Clinical Characteristics

**Figure 2:**
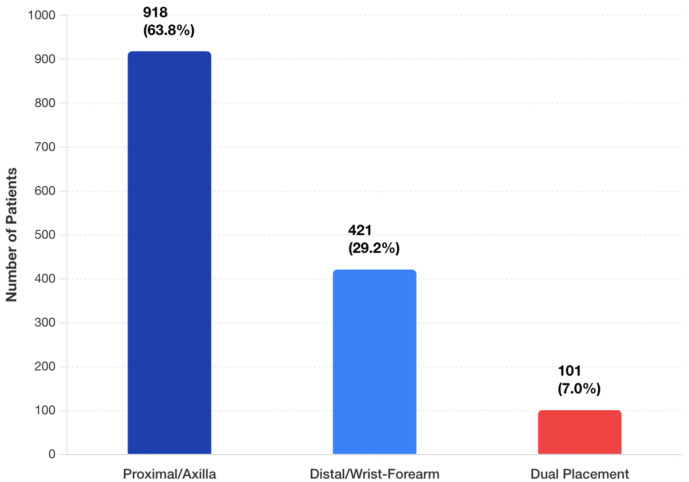
Distribution of BCRL patients by VLNT placement strategy (*n* = 1440).

Patient demographics were comparable between patients who underwent a VLNT with a proximal and a distal recipient site (Table 1). Of the 1440 patients, the majority had proximal placement of the VLNT (*n* = 918, 63.8%) with fewer receiving distal (*n* = 421, 29.2%) placement. Dual placement (VLNTs placed both proximally and distally) was uncommon, reported in only 101 patients (7.0%) (Figure 2). Patient age and BMI were similar between all three groups. A greater proportion of patients with proximal VLNT received adjuvant radiation (85%) compared to distal (70%) or dual placement (75%) cohorts.

**Figure 3:**
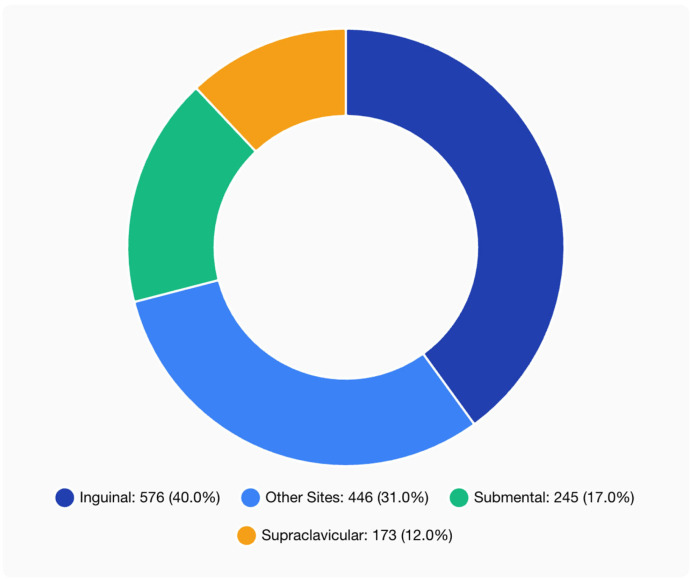
Donor site utilization in BCRL VLNT procedures.

Donor site distribution included inguinal (576 patients, 40.0%), submental (245 patients, 17.0%), supraclavicular (173 patients, 12.0%), and other locations (446 patients, 31.0%), (Figure 2). Common recipient vessels were the thoracodorsal (proximal) and the radial artery (distal). End-to-end anastomosis predominated across all groups (75% in proximal, 65% in distal, and 59% in dual placement) with higher rates of end-to-side and flow-through techniques in distal and dual-placement VLNTs (Figure 3). Supercharging was rare, reported in 5.8% of cases (84 patients total).

### 3.3. Complications

Across 1440 pooled patients, minor complications (e.g., seroma, minor wound infection, donor site lymphocele) occurred in about 18% of cases, and major complications in about 5%—and these rates did not significantly differ by recipient site (proximal vs. distal). The incidence of total flap loss (complete flap failure) was very low, on the order of only ~1–2% in the literature [45].

### 3.4. Clinical Outcomes by Placement Strategy

**Figure 4:**
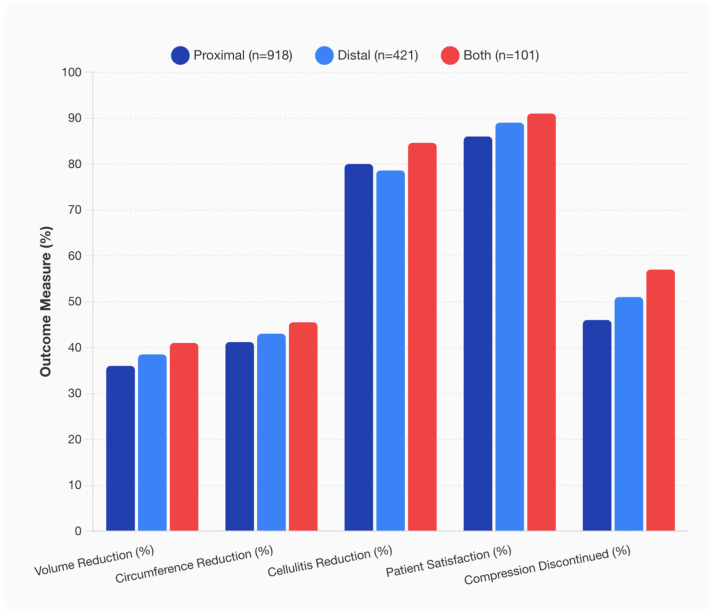
Comparative clinical outcomes by VLNT placement strategy.

VLNTs significantly reduced upper extremity volume across all groups. Mean volume reductions were 36% (proximal), 38% (distal), and 41% (dual placement). Mean circumference reduction rates were similarly favorable, at 41%, 43%, and 45%, respectively. Cellulitis episodes decreased by 80% (proximal), 79% (distal), and 84% (dual site). Patient satisfaction was 86% in the proximal group, 89% in the distal group, and 91% in the dual placement cohort. Rates of compression garment discontinuation were 46% for proximal, 51% for distal, and 57% for dual placement. None of these between-group differences reached statistical significance (all *p* > 0.30) (Figure 4).

### 3.5. Scar Release + VLNT

**Figure 5:**
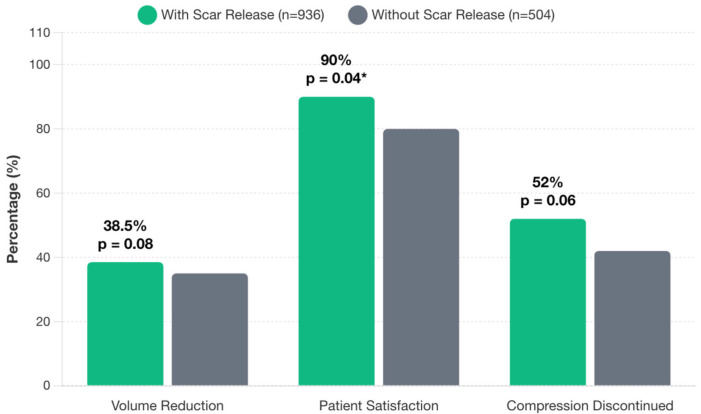
Impact of concurrent scar release on VLNT outcomes.

Scar release was reported in 936 patients (65%), most frequently with an axillary recipient site. Patients who underwent scar release reported significantly higher subjective improvement rates (90%) compared to those who did not (80%) (*p* = 0.04). Volume reduction was 38.5% among patients with scar release compared to 35.0% in those without, though this difference approached but did not reach statistical significance (*p* = 0.08). Compression garment discontinuation showed a trend toward improvement with scar release (52% vs. 42%, *p* = 0.06) (Figure 5).

### 3.6. Supercharging of VLNT

**Figure 6:**
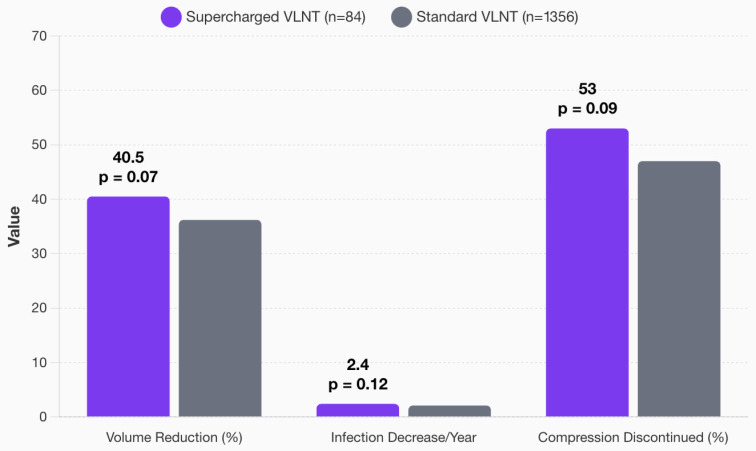
Venous supercharging in VLNT: impact on clinical outcomes.

Among the 84 patients who underwent supercharged VLNT (5.8% of total cohort), the mean volume reduction was greater than the standard VLNT group (40.5% vs. 36.2%, *p* = 0.07). Infection rate also trended favorably for supercharging but was not statistically significant with 2.4 vs. 2.1 infections/year (*p* = 0.12). Finally, improvements in compression were noted in the supercharged cohort, with discontinuation in 53% in supercharged VLNTs compared to 47% in standard VLNTs (*p* = 0.09) (Figure 6).

### 3.7. Timing of VLNT

**Figure 7:**
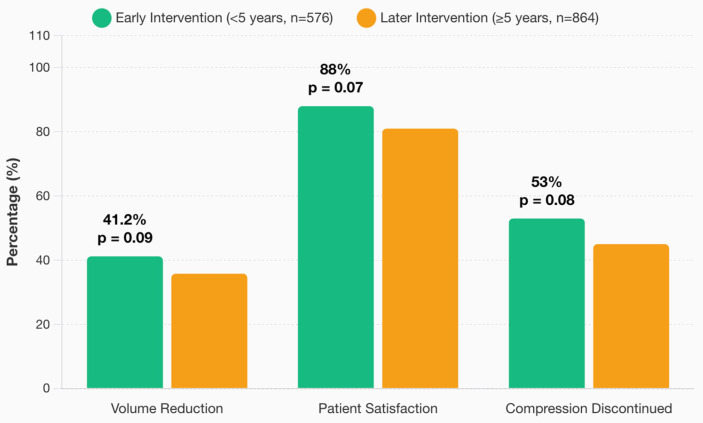
Temporal factors in VLNT outcomes: early vs. delayed intervention.

Early surgical intervention (<5 years from diagnosis, *n* = 576) was associated with greater volume reduction (41.2%) compared to later intervention [(≥5 years, *n* = 864, 35.8%; (*p* = 0.09)]. Patient satisfaction was 88% for early intervention vs. 81% for later intervention (*p* = 0.07). Compression garment discontinuation was 53% for early intervention vs. 45% for later intervention (*p* = 0.08) (Figure 7).

### 3.8. Comprehensive Outcome Analysis

Our multidimensional outcome profile analysis demonstrated that dual placement strategies showed consistently superior outcomes across all five measured parameters (volume reduction, circumference reduction, cellulitis control, patient satisfaction, and compression freedom), though individual differences did not reach statistical significance. Longitudinal analysis over 12 months revealed progressive volume reduction across all placement strategies, with dual placement maintaining consistent superiority throughout the follow-up period. The parallel trajectories suggest that placement-related advantages are established early and maintained over time. At 12 months, volume reduction reached approximately 41% for proximal, 43% for distal, and 45.5% for dual placement, maintaining the between-group differences observed throughout follow-up.

## 4. Discussion

Breast cancer-related lymphedema is a significant, life-altering complication for breast cancer survivors, affecting 20–30% of patients who undergo an axillary lymph node dissection (ALND) and/or radiation therapy [2,46]. As surgical strategies for lymphedema continue to evolve, vascularized lymph node transfers (VLNTs) are gaining widespread adoption as a physiologic alternative for patients when a lymphovenous bypass alone is insufficient [47,48]. Despite increasing clinical use of VLNTs, there is a paucity of high-level evidence guiding specific nuances of the surgical procedure, including recipient site selection, necessity of scar release, and benefits of supercharging techniques. Therefore, we conducted a meta-analysis and systematic review to evaluate the role of VLNT recipient site selection and its nuances in patients undergoing treatment of BCRL.

In our study, we reviewed the outcomes of 37 studies (1440 patients), and our results indicate that VLNTs significantly reduced upper extremity volume across all groups: 36% (proximal), 38% (distal), and 41% (dual placement). Similarly, all three recipient sites led to similar outcomes with respect to circumference reduction, excess limb volume reduction, incidence of infections, discontinuation of compression, and patient satisfaction. This finding indicates that VLNTs, regardless of recipient sites, are efficacious in the treatment of BCRL. Of the 1440 patients, the majority of patients had proximal placement of the VLNT in the axilla (63.8%), which is most likely due to the physiologic rationale of directly addressing the cause of the lymphatic obstruction by promoting lymphangiogenesis and creating new afferent and efferent lymphatics at the site of initial lymphatic disruption [45,49]. Further, a proximal recipient site allows for concurrent scar release in the setting of radiation fibrosis or surgical scarring, which was observed in 65% of the study population [30,46]. These patients reported overall subjective and volumetric improvement, and compression garment discontinuation [30,46]. The value and risks of scar release, however, differ depending on the quality of the soft tissues; while a denser scar yields greater benefit from release with the addition of vascularized tissue, dissection through dense scar and fibrotic tissues is technically challenging and tedious, with risk of damaging large vessels or functional nerves in the axilla [8].

While we found that all three recipient locations led to similar outcomes statistically, dual-site VLNTs did achieve a slightly higher rate of mean volume reduction, patient satisfaction, and rates of compression garment discontinuation. This finding can be secondary to the smaller sample size of dual-site VLNT (101 patients) and may indicate the evolving role of this procedure in the treatment of BCRL patients. Dual-site VLNT capitalizes on the distinct advantages of both recipient sites, restoring flow at the site of lymphatic disruption and enhancing distal shunting and drainage [9,50,51]. In addition to the benefits of proximal VLNTs, dual-site VLNTs allow for the bypassing of inefficient lymphatic system at the forearm due to lymphatic loss of contractility, alterations of the valvular system, and changes in the collecting ducts, which together result in distal lymphatic pump failure [50]. Therefore, this technique offers unique advantages of ability to perform scar release at the axilla, distal placement of VLNT at the site of maximal pooling such as forearm or wrist, and proximal placement of VLNT to promote lymphangiogenesis. While the results are promising, this technique is more technically challenging as it requires multiple lymph node flaps, which are typically harvested intra-abdominally using laparoscopic, robotic, or open approaches. In addition, we need to approach this technique with caution as the sample size of this study population is smaller than other types of techniques.

In addition to evaluating the role of the recipient site, we analyzed the timing of VLNT and the efficacy of supercharging VLNT. We found that the timing of VLNT plays a significant role in the overall outcome. Patients who underwent early surgical intervention (<5 years from diagnosis) had a greater volume reduction, patient satisfaction, and compression garment discontinuation. While statistical significance was not achieved, all measurements approached statistical significance. These findings suggest that prompt referral before irreversible lymphatic damage may optimize surgical outcomes and support early intervention paradigms. Similarly, we found that supercharging VLNT is an important consideration in preoperative planning. The physiological premise for venous supercharging is that that a single venous outflow may be insufficient, leading to venous hypertension and compromising both flap perfusion and lymphatic function [52]. In our pooled analysis, supercharged VLNTs with dual venous outflows or intra-flap fistula creation were used to rebalance these hemodynamics. The limited number of studies reporting supercharging limits our ability to draw definitive conclusions, but the consistency of positive trends in improved limb circumference reduction (*p* = 0.07) and discontinuation of compression (*p* = 0.09) with supercharging underscores the need for further investigation. Standardized prospective randomized trials are necessary to better understand the role of supercharging in VLNTs, controlling for donor and recipient sites.

Our review highlights important limitations in the available VLNT literature. First, the majority of the reported data are based on retrospective studies with a lack of standardization, which may have implications on validity, reliability, and interpretability of findings due to biases (selection, recall, reporting). The limited number of prospective studies and small sample sizes hinder generalizability of findings. Second, while our meta-analysis includes a large, pooled sample, subgroup analyses were underpowered due to small sample sizes. Third, data were amalgamated across studies that used varied definitions and metrics for lymphedema. These limitations highlight the need for well-designed, multi-institutional prospective studies to establish evidence-based guidelines for VLNT techniques. Lastly, the recipient site selected was based on surgeon preference rather than comparative design in most studies. As such, results are largely representative of lymphedema outcome differences “between studies” rather than “within studies”. These data may therefore be confounded by differences in flap donor site, surgical techniques for dissection and anastomosis, severity of lymphedema treated, and methods of outcome assessment. These limitations underscore a need for well-designed, multi-center, prospective trials with head-to-head comparisons of recipient sites, standardized outcome definitions and follow-up intervals, targeted evaluation of dual-placement VLNT, rigorous study of supercharging strategies, and structured perioperative rehabilitation protocols.

Lastly, our study supports that VLNT is effective for the treatment of BCRL, regardless of its placement. In the absence of statistically significant site-based efficacy differences, recipient site selection may be individualized according to anatomy and vessel availability, soft-tissue quality and scarring including radiation changes, and patient preference. In addition, patients should undergo early surgical intervention to maximize the overall outcome.

## 5. Conclusions

Vascularized lymph node transfer is an effective surgical intervention for patients with BCRL, offering improvements in limb volume, frequency of cellulitis, and patient-reported outcomes across placement strategies. Our analysis supports that VLNT placement, whether proximal or distal, does not significantly alter clinical outcomes. We identified no difference in circumference reduction, volume reduction, rate of infection, postoperative use of compression, patient-reported outcomes, or rate of complications. Dual placement may be an effective strategy to capitalize on the benefits of both proximal and distal placement; however, the clinical value of dual-placement VLNT requires further investigation with prospective studies using standardized outcome measures and longer follow-up periods. The lack of significant difference in clinical outcomes between placement locations, as indicated by this meta-analysis, suggests other factors such as recipient vessel availability, soft-tissue quality, cosmesis, and patient preference may play more critical roles in determining surgical success. VLNT adjuncts such as scar release and supercharging appear to further enhance patient outcomes.

## Figures and Tables

**Table 1:** Demographic and clinical characteristics of patients with breast cancer-related lymphedema undergoing vascularized lymph node transfer (*n* = 1440).

Characteristic	Total (*n* = 1440)	Proximal (*n* = 918)	Distal (*n* = 421)	Both (*n* = 101)
**Demographics**				
Age, y, mean ± SD	54.0 ± 8.2	55.2 ± 8.5	53.8 ± 7.8	54.5 ± 8.0
Range	16–80	18–80	16–78	22–75
BMI, kg/m^2^, mean ± SD	27.1 ± 4.2	27.5 ± 4.3	27.0 ± 4.0	27.2 ± 4.1
Range	22.5–33.0	22.8–33.0	22.5–32.5	23.0–32.0
Radiation therapy, *n* (%)	1080 (75.0)	780 (85.0)	294 (69.8)	76 (75.2)
Time to VLNT, y, mean ± SD	6.0 ± 3.5	6.2 ± 3.6	5.8 ± 3.4	5.9 ± 3.5
**VLNT Donor Site, *n* (%)**				
Inguinal	576 (40.0)	414 (45.1)	147 (34.9)	15 (14.9)
Submental	245 (17.0)	138 (15.0)	84 (20.0)	23 (22.8)
Supraclavicular	173 (12.0)	101 (11.0)	59 (14.0)	13 (12.9)
Other ^a^	446 (31.0)	265 (28.9)	131 (31.1)	50 (49.5)
**Surgical Technique**				
**Recipient vessels, *n* (%)**				
Thoracodorsal	—	643 (70.0)	—	— ^b^
Radial	—	—	211 (50.1)	— ^b^
Ulnar/other	—	275 (30.0)	210 (49.9)	— ^b^
**Anastomosis type, *n* (%)**				
End-to-end	1022 (71.0)	689 (75.1)	274 (65.1)	60 (59.4)
End-to-side	274 (19.0)	138 (15.0)	105 (24.9)	31 (30.7)
Flow-through	144 (10.0)	91 (9.9)	42 (10.0)	10 (9.9)
**Adjunct procedures, *n* (%)**				
Scar release	936 (65.0)	734 (80.0)	126 (29.9)	76 (75.2)
Supercharging	84 (5.8)	46 (5.0)	29 (6.9)	9 (8.9)

BMI = body mass index; VLNT = vascularized lymph node transfer. Proximal = axillary placement; distal = wrist/forearm placement; both = dual placement. ^a^ Includes gastroepiploic, jejunal mesenteric, lateral thoracic, and omental lymph nodes. ^b^ Mixed vessels used for dual placement.

## Data Availability

All data analyzed in this study are available in the published literature referenced in this manuscript.

## Supplementary Materials

The following supporting information can be downloaded at: https://www.mdpi.com/article/10.3390/jcm14207281/s1, Table S1: PRISMA 2020 Checklist.

## Abbreviations

The following abbreviations are used in this manuscript:

ALND	Axillary lymph node dissection
BCRL	Breast cancer-related lymphedema
BMI	Body mass index
DIEP	Deep inferior epigastric artery perforator
LVA	Lymphovenous anastomosis
LVB	Lymphovenous bypass
MLD	Manual lymphatic drainage
pLVB	Prophylactic lymphovenous bypass
PRISMA	Preferred Reporting Items for Systematic Reviews and Meta-Analyses
VLNT	Vascularized lymph node transfer
