# Supplementary material for: Proximal vs. Recipient Site for Vascular Lymph Node Transfers for Breast Cancer-Related Lymphedema: A Meta-Analysis and Systematic Review
Source: J Clin Med. 2025 Oct 15;14(20):7281. doi: 10.3390/jcm14207281 (PMC12565181; doi:10.3390/jcm14207281)
Supplement: Supplementary file 1 [file jcm-14-07281-s001.zip › jcm-3832512-supplementary.pdf]

Supplementary Figure S1. PRISMA 2020 Checklist

Section and Topic	Item #	Checklist item	Location where item is reported
TITLE	TITLE	TITLE	
Title	1	Identify the report as a systematic review.	Title page & Article Type: identified as a systematic review and meta-analysis.
ABSTRACT	ABSTR ACT	ABSTRACT	
Abstract	2	See the PRISMA 2020 for Abstracts checklist.	Abstract: Structured, with background/objectives, methods (search of PubMed, MEDLINE, Embase; 2015-2025), results, conclusions.
INTRODUCTION	INTROD UCTION	INTRODUCTION	
Rationale	3	Describe the rationale for the review in the context of existing knowledge.	Introduction (§1): Rationale for recipient-site selection in VLNT and evidence gaps for BCRL.
Objectives	4	Provide an explicit statement of the objective(s) or question(s) the review addresses.	Introduction (§1, closing): Objective to compare VLNT placement (proximal vs. distal vs. dual) and adjuncts (scar release, supercharging, timing).
METHODS	METHO DS	METHODS	
Eligibility criteria	5	Specify the inclusion and exclusion criteria for the review and how studies were grouped for the syntheses.	§2.2 Eligibility Criteria: PICOS; human BCRL; clinical designs (RCTs, cohorts, case-control, observational, larger case series); quantitative outcomes required.
Information sources	6	Specify all databases, registers, websites, organisations, reference lists and other sources searched or consulted to identify studies. Specify the date when each source was last searched or consulted.	§2.3 Information Sources & Search Strategy: PubMed (MEDLINE), Ovid MEDLINE, Embase; comprehensive search performed April-May 2025; study window 2015-Mar 1, 2025.
Search strategy	7	Present the full search strategies for all databases, registers and websites, including any filters and limits used.	§2.3 Information Sources & Search Strategy: Full strategies provided in Supplementary Materials (per manuscript: Supplementary Figure S1 Supplementary Figure S1), PRISMA-S guided keywords/MeSH.
Selection process	8	Specify the methods used to decide whether a study met the inclusion criteria of the review, including how many reviewers screened each record and each report retrieved, whether they worked independently, and if applicable, details of automation tools used in the process.	§2.4 Study Selection (Screening & Exclusion): Two independent screeners; PRISMA flow (Figure 1). 844 unique records screened; 690 excluded at title/abstract; reasons listed.
Data collection process	9	Specify the methods used to collect data from reports, including how many reviewers collected data from each report, whether they worked independently, any processes for obtaining or confirming data from study investigators, and if applicable, details of automation tools used in the process.	§2.5 Data Collection and Extraction: Two independent extractors; standardized form; cross-verification; no automation tools.
Data items	10a	List and define all outcomes for which data were sought. Specify whether all results that were compatible with each outcome domain in each study were sought (e.g. for all measures, time points, analyses), and if not, the methods used to decide which results to collect.	§2.6 Outcomes Assessed: Volume/circumference reduction; cellulitis frequency; compression discontinuation; patient-reported satisfaction; complications; follow-up duration.

Section and Topic	Item #	Checklist item	Location where item is reported
Data items	10b	List and define all other variables for which data were sought (e.g. participant and intervention characteristics, funding sources). Describe any assumptions made about any missing or unclear information.	§§2.5-2.6 Data Items: Demographics; surgical details; adjuncts; unit harmonization to % reduction; assumptions noted as needed.
Study risk of bias assessment	11	Specify the methods used to assess risk of bias in the included studies, including details of the tool(s) used, how many reviewers assessed each study and whether they worked independently, and if applicable, details of automation tools used in the process.	Title page & Article Type: identified as a systematic review and meta-analysis.
Effect measures	12	Specify for each outcome the effect measure(s) (e.g. risk ratio, mean difference) used in the synthesis or presentation of results.	Title page & Article Type: identified as a systematic review and meta-analysis.
Synthesis methods	13a	Describe the processes used to decide which studies were eligible for each synthesis (e.g. tabulating the study intervention characteristics and comparing against the planned groups for each synthesis (item #5)).	§2.8 Synthesis Eligibility: Studies included in each synthesis if they reported the outcome of interest.
Synthesis methods	13b	Describe any methods required to prepare the data for presentation or synthesis, such as handling of missing summary statistics, or data conversions.	§2.8 Data Preparation: Harmonized units and summarized continuous data to common metrics.
Synthesis methods	13c	Describe any methods used to tabulate or visually display results of individual studies and syntheses.	§2.8 Presentation: Tables/figures summarize study characteristics and pooled outcomes; PRISMA flow Figure 1.
Synthesis methods	13d	Describe any methods used to synthesize results and provide a rationale for the choice(s). If meta-analysis was performed, describe the model(s), method(s) to identify the presence and extent of statistical heterogeneity, and software package(s) used.	§2.8 Models/Heterogeneity/Software: Random-effects (DerSimonian-Laird); heterogeneity via Q and I^2; R (v4.2.2, meta package).
Synthesis methods	13e	Describe any methods used to explore possible causes of heterogeneity among study results (e.g. subgroup analysis, meta-regression).	§2.8 Subgroups: Prespecified-recipient site (proximal, distal, dual); scar release; supercharging; timing.
Synthesis methods	13f	Describe any sensitivity analyses conducted to assess robustness of the synthesized results.	§2.8 Sensitivity: Limited to model checks; no formal sensitivity analyses beyond subgrouping.
Reporting bias assessment	14	Describe any methods used to assess risk of bias due to missing results in a synthesis (arising from reporting biases).	Title page & Article Type: identified as a systematic review and meta-analysis.
Certainty assessment	15	Describe any methods used to assess certainty (or confidence) in the body of evidence for an outcome.	Title page & Article Type: identified as a systematic review and meta-analysis.
RESULTS	RESULTS	RESULTS	
Study selection	16a	Describe the results of the search and selection process, from the number of records identified in the search to the number of studies included in the review, ideally using a flow diagram.	Results §3.1 & Figure 1: Records identified, screened, excluded (with counts), and included (n~37 studies).
		explain why they were excluded.	
Study characteristics	17	Cite each included study and present its characteristics.	Title page & Article Type: identified as a systematic review and meta-analysis.
Risk of bias in studies	18	Present assessments of risk of bias for each included study.	Title page & Article Type: identified as a systematic review and meta-analysis.

Section and Topic	Item #	Checklist item	Location where item is reported
Results of individual studies	19	For all outcomes, present, for each study: (a) summary statistics for each group (where appropriate) and (b) an effect estimate and its precision (e.g. confidence/credible interval), ideally using structured tables or plots.	Title page & Article Type: identified as a systematic review and meta-analysis.
Results of syntheses	20a	For each synthesis, briefly summarise the characteristics and risk of bias among contributing studies.	Results overview & §4 Discussion: Characteristics and limitations of contributing evidence considered.
Results of syntheses	20b	Present results of all statistical syntheses conducted. If meta-analysis was done, present for each the summary estimate and its precision (e.g. confidence/credible interval) and measures of statistical heterogeneity. If comparing groups, describe the direction of the effect.	Results §§3.4-3.8: Statistical synthesis results; heterogeneity addressed per §2.8 (I^2).
Results of syntheses	20c	Present results of all investigations of possible causes of heterogeneity among study results.	Results §§3.4-3.8: Subgroup findings exploring heterogeneity (site, scar release, supercharging, timing).
Results of syntheses	20d	Present results of all sensitivity analyses conducted to assess the robustness of the synthesized results.	Not applicable-no additional sensitivity analyses beyond subgroup/model checks (§2.8).
Reporting biases	21	Present assessments of risk of bias due to missing results (arising from reporting biases) for each synthesis assessed.	Abstract: Structured, with background/objectives, methods (search of PubMed, MEDLINE, Embase; 2015-2025), results, conclusions.
Certainty of evidence	22	Present assessments of certainty (or confidence) in the body of evidence for each outcome assessed.	Abstract: Structured, with background/objectives, methods (search of PubMed, MEDLINE, Embase; 2015-2025), results, conclusions.
DISCUSSION	DISCUSSION	DISCUSSION	
Discussion	23a	Provide a general interpretation of the results in the context of other evidence.	§4 Discussion: Interpretation in context of prior evidence; implications for VLNT site selection.
Discussion	23b	Discuss any limitations of the evidence included in the review.	§4 Discussion: Limitations of included studies (design, sample size, measurement variability).
Discussion	23c	Discuss any limitations of the review processes used.	§4 Discussion: Limitations of review process (no formal RoB tool; underpowered subgroups).
Discussion	23d	Discuss implications of the results for practice, policy, and future research.	§4 Discussion: Practice implications and need for standardized, prospective multi-center trials.
OTHER INFORMATION	OTHER INFORMATION	OTHER INFORMATION	
Registration and protocol	24a	Provide registration information for the review, including register name and registration number, or state that the review was not registered.	§2.1 Protocol/Registration: Not registered on PROSPERO or other registry.
Registration and protocol	24b	Indicate where the review protocol can be accessed, or state that a protocol was not prepared.	§2.1 Protocol: No prior protocol document prepared.
Registration and protocol	24c	Describe and explain any amendments to information provided at registration or in the protocol.	§2.1 Amendments: Not applicable (no registered protocol).

Section and Topic	Item #	Checklist item	Location where item is reported
Support	25	Describe sources of financial or non-financial support for the review, and the role of the funders or sponsors in the review.	Abstract: Structured, with background/objectives, methods (search of PubMed, MEDLINE, Embase; 2015-2025), results, conclusions.
Competing interests	26	Declare any competing interests of review authors.	Abstract: Structured, with background/objectives, methods (search of PubMed, MEDLINE, Embase; 2015-2025), results, conclusions.
Availability of data, code and other materials	27	Report which of the following are publicly available and where they can be found: template data collection forms; data extracted from included studies; data used for all analyses; analytic code; any other materials used in the review.	Abstract: Structured, with background/objectives, methods (search of PubMed, MEDLINE, Embase; 2015-2025), results, conclusions.
